# The Hospital. Nursing Section

**Published:** 1903-05-23

**Authors:** 


### The Hospital.

### Nursing Section.

Contributions for this Section of "The Hospital" should be addressed to the Editor, "The Hospital" Nursing Section, 28 & 29 Southampton Street, Strand, London, W.C.

No. 869.—Vol. XXXIV. *SATURDAY, MAY* 23, 1903.

### Notes on News from the Nursing World.

### THE QUEEN AND THE EDINBURGH NURSES.

There were no fewer than sixty members of the nursing staff of the old Edinburgh City Hospital present last week at the opening by the King and Queen of the new building at Collinton Mains, a suburb of the capital. The committee considerately arranged that they should have a very good and prominent place. They stood in double line and curtsied as the Royal carriage passed, the Queen taking special notice of them. In the room prepared for their Majesties the lady superintendent, Miss E. C. Sandford, was accompanied by the two assistant matrons, Miss Armitstead and Miss Priest, and six ward sisters, who handed tea to the King and Queen and their suite. The Queen was graciously pleased to accept a bouquet of roses and lilies of the valley from the nurses and was evidently gratified with the gift. When the lady superintendent, by whom, with the medical superintendent, the King and Queen were received, had been presented to the Queen, she had the opportunity of telling her how greatly her Majesty's interest in nurses is appreciated in Scotland, and the Queen then asked Miss Sandford many questions about the new hospital, expressing her admiration at its size and the magnificence of the position.

### ROYALTY IN A NURSE'S COTTAGE.

On the occasion of the inauguration of the electric tramway between Westminster and Tooting, last Friday, the Prince and Princess of Wales visited the home of a nurse in the houses of the London County Council, on the Totterdown Estate. Mrs. Hutchinson, whose maiden name was Pilkington, received part training at the London Hospital, Whitechapel, 12 years ago, and afterwards was awarded a maternity certificate at the City of London Hospital, City Road. She was the first district nurse at Kew, where she worked for nearly three years, and subsequently took up private nursing. Mrs. Hutchinson had the honour of being introduced to the Prince and Princess of Wales, by Lord Carrington, and they remained some time in her nicely-furnished little home, asking questions and taking a kindly interest in all they saw.

### COLONIAL NURSING ASSOCIATION.

The annual meeting of the Colonial Nursing Association will take place at Chelsea Hospital on Thursday, June 11th. Princess Henry of Battenberg will be present at the meeting, over which Lord Grey will preside. The speakers will include Lord Selborne and Sir Harry Johnston.

### CENTRAL MIDWIVES BOARD.

We understand that three meetings of the Central Midwives Board have been held this month, and that the drafting of the rules under Section 31 of the Midwives Act, 1902, having been completed, the secretary has forwarded a copy to the Privy Council for their approval, together with some draft "suggestions to County Councils."

### LONDON SCHOOL NURSES' SOCIETY.

An important meeting on behalf of the London School Nurses' Charity will be held, by permission of Lady Windsor, at 54 Mount Street, on Tuesday next at 3.30 p.m. The speakers will be Lady Jeune, Sir Henry Burdett, K.C.B., Mr. Evelyn Cecil, M.P., and the Hon. E. Lyulph Stanley. We hope that the interest excited will be considerable, and that the funds of this most admirable movement will receive the substantial addition required in order to extend its operations to anything like the necessary degree. An excellent idea of the work done is given in our columns to-day by a nurse who is engaged in it.

### SCHOOL NURSES IN THE STATES.

The Health Department of New York has assigned nurses to 39 public schools in Manhattan. Necessary supplies have been furnished to the schools, and principals have been invited to give the nurses as much assistance as possible. The nurses are to examine the children as to cleanliness, and to instruct the mothers at their homes how to treat all cases of sickness. The schools selected are in the congested districts of the east and west sides of Manhattan, and the authorities are warmly in favour of the arrangement.

### NIGHT NURSING IN WORKHOUSE INFIRMARIES.

In the paper read by Dr. Arthur Downes, of the Local Government Board, at the West Midland Poor Law Conference last week, on "Nursing in Workhouses and the Report of the Departmental Committee," he strongly urged that the permanent staff of a workhouse infirmary should be adequate to cope with night nursing, and that extra nurses should be obtained from an institution when necessary. He rightly says, "It is not enough—it is dangerous—to rely on attendants who are to call someone else," and "moreover, in practice, the attendant may think twice before calling anyone." Before passing away from this subject Dr. Downes asked his audience at Malvern to picture what was meant by a long night of suffering with no hand on which to depend for relief in its simplest forms. He thought that they would agree with him "that were there but one nurse in the world, she should be placed on night duty."

### UNIVERSITY COLLEGE HOSPITAL.

The annual meeting of University College Hospital Ladies' Association was held in the board-room of the hospital on Monday afternoon, the Duchess of Devonshire presiding. The adoption of the report, which stated that the association at present consists of 344 members, was moved by Lady Parker and seconded by Lady Russell Reynolds. It was intimated that the association propose to continue the donation of £100 which was given last year to maintain the "University College Hospital Ladies' Association bed" in the hospital. The executive committee having been elected, Miss Finch, matron of the hospital, thanked the members for the garments which had been sent in, and for the supply of eggs, flowers and fruit.

### THE NEW MATRON OF CROYDON INFIRMARY.

ON Tuesday the Croydon Board of Guardians met to consider applications for the post of matron and superintendent of nurses under the reorganisation scheme. Four candidates were selected to appear before the Board, and the choice fell by a substantial vote upon Miss Annie Winifred Pagen, senior assistant matron at the Poplar and Stepney Sick Asylum, Bromley-by-Bow. Miss Pagen's selection was warmly supported on medical grounds by Dr. W. B. Addison, a member of the Board. The new matron was trained at Brownlow Hill Infirmary, Liverpool, and after her training joined the nursing staff of the Poplar and Stepney Asylum, and was promoted from one position to another until she became senior assistant matron. The Croydon Guardians have submitted to the Local Government Board for their approval a scheme drawn up by the medical superintendent, which, if adopted, will result in the employment of the following nursing staff:—Four sisters or head nurses at a commencing salary of £30 rising to £35; a night superintendent with a commencing salary of £35 rising to £40; 40 probationers to receive £6 the first year, £12 the second, and £18 the third; a midwife with a commencing salary of £35 rising to £40, and an assistant midwife with a salary of £18. There will also be three female and three male lunatic attendants.

### CALCUTTA HOSPITAL NURSES' INSTITUTION.

WE have received a copy of the report of the Calcutta Hospital Nurses' Institution for 1903. It is now responsible for the payment of nearly a hundred nurses in the different Calcutta hospitals, namely, the Presidency General, the Medical College, and the Eden. As to the first of these, the staff consists of 29 senior and 22 junior nurses, two probationers, and an extra nurse. Owing to the absence of Major Pilgrim, surgeon-superintendent during the year, no report on the work of this hospital is given, but the second resident surgeon states that so far as the nursing arrangements came under his notice they were satisfactory. Major Pilgrim's substitute might, however, have issued a report. At the Medical College Hospital, where the establishment consists of a matron, 21 senior and 11 junior nurses, two probationers, and a surgical nurse, Colonel Bomford reports that "the nurses have given every satisfaction at the hospital attached to this college, and all we want is more of them. We are working with what may be described as the minimum number of nurses that will ensure every patient receiving some attention from an attendant better than an ordinary coolie, but at night one nurse may have as many as 66 patients to attend to, and this is obviously too many." At the Eden Hospital the staff comprises a matron, four ward nurses, and two surgical nurses. Colonel Peck in his report "has nothing but praise for the nursing staff." Here the night nursing is done by senior pupils, the ward nurses being in charge both by day and night. This, Colonel Peck affirms, is an intolerable strain, and he says it is imperative that a night superintendent should be appointed so that the ward nurses may get their night undisturbed. It is hoped by the manager of the Institution that the funds will permit of an appointment being made at an early date.

### THE NURSES OF FOLKESTONE HOSPITAL.

LAST week Mrs. Lenington, wife of the visiting surgeon, gave an "at home" to the nurses of Folkestone Hospital and a number of friends. Considerable amusement was caused by each guest bringing some article costing a penny only, and wonderful was the collection. Mrs. Tyson was voted to have brought the most useful article, namely, an enamel saucepan. In a few well-chosen words, Mrs. Tyson expressed her admiration for the good work done by the nurses. She then presented the prizes, which had been given by Mr. Lenington, the first to Nurse Coxter, the second to Nurse Lyons and Nurse Rant, both being equally good, and the third to Nurse Armstrong. The nurses enjoyed themselves thoroughly, and went back to the hospital feeling rested and cheered by the kindness and hospitality of their surgeon and his wife. Mrs. Lenington held the post of theatre sister at St. Mary's Hospital, Paddington, before her marriage.

### BELFAST GUARDIANS AND OUTSIDE NURSES.

THE question of allowing outside nurses to receive fever training in the Belfast Fever Hospital has been under the consideration of the Board of Guardians. This is not the first time that the issue has been raised. Two years ago the manager of the Mater Infirmorum Hospital asked the guardians to allow the admission of one or two of their nurses, but the request was refused on the ground of the large number of nurses in the home requiring fever training. The explanation was accepted as satisfactory. Since then, however, the state of affairs has confessedly altered, and Dr. Robb, the medical superintendent, lately informed one of the guardians that he required more nurses for fever training, and that the admission of a couple of Mater nurses and the supernumeraries would meet the case, and be in addition a benefit to the fever hospital itself. Mr. O'Hare, the guardian referred to, brought the matter before the Board the other day, but was only supported by two of his colleagues. Unhappily, other members introduced religion into the discussion, and, oblivious of the fact that the Mater Hospital is partly staffed by lay nurses, Anglican as well as Romanist, treated the proposal as if the object in view were, by means of a side-wind, to add Sisters of Mercy to the nursing staff of the infirmary. It is, of course, open to any hospital to decline to receive outside nurses for training, but their exclusion on religious grounds is as mistaken as it is indefensible.

### PROGRESS AT WOOLWICH.

THE annual report of the Woolwich and District Nursing Association affords gratifying proof of the fact that the working classes in Woolwich not only appreciate the work, but are glad to afford financial support to it. The chairman, in his speech, mentioned that upwards of 140 bodies were contributing to the funds; and a feature of the report is that a demonstration of the local trades unions, friendly and temperance societies, yielded the handsome sum of £40 16s. 6d. For the general account there was a balance of £35 11s. 6d. in hand, and in the endowment fund account the balance to the good is £429 18s. 5d. The meeting attracted a large attendance, and the increasing prosperity of the organisation is evidently due to the practical interest taken by all classes in its operations.

### A BITTER CRY FROM BRISTOL.

THE withdrawal by the Bristol District Nurses' Society of one of their staff from a densely populated region known as Moorfields, Redfield, and Pilemarsh, has provoked a bitter cry from the localities affected. The explanation of the action of the Society relieves it of all blame. A nurse has been working in the district for 20 years, and, owing to the growth of population, it has at last become more than she can manage. The Society has therefore been compelled to restrict her area of duty, and it does not possess the funds to put on an additional nurse for the district cut off. We can quite understand the view of one of the clergy, that the withdrawal of a nurse from a poor district which has grown accustomed to her devoted ministration causes a much more poignant sense of suffering than would be felt if the nurse had never been at work. But there is only one possible remedy. The cost of another nurse is about £70 a year. It is incredible that, if the facts of the case are put before the wealthy and well-to-do people of Bristol, they will decline to supply the money required. In common fairness to them, we are bound to assume that so far they have not been made aware of the pressing needs of the sick poor at their own door.

### WARWICK UNION INFIRMARY.

THE Local Government have sanctioned the appointment of a head nurse at the new infirmary of the Warwick Union, instead of a superintendent nurse, so long as Mrs. Crosland remains workhouse matron and so long as the arrangement proves satisfactory. This is as it should be. Mrs. Crosland is a trained nurse, and fully competent to undertake the superintendence of the nursing staff.

### AUCKLAND WORKING MEN'S NURSING ASSOCIATION.

IT will be remembered that a few months ago a number of the working men of Bishop Auckland irate, not only at the conversion of the District Nursing Association into a provident organisation but at the dismissal of Miss Wood, who has been Queen's Nurse for eight years, determined to start an association of their own. We do not understand how the provident organisation, which, while requiring a fee for the services of the nurse, still claims to be a branch of the Queen's Jubilee Institute, can justify, or is permitted to use, its title. But we learn that the new nursing movement is making excellent progress. Its rules are simple, and the object is clearly defined, namely, to provide trained nurses to nurse the sick poor and industrial classes in their own homes. All subscribers of 2s. a year and upwards are entitled to attend and vote at annual and special meetings. We should be sorry to see such splits as this repeated elsewhere, but the District Association, in departing from its original principles and starting on an entirely fresh basis, practically invited a fresh effort to provide for the nursing of the sick poor without money and without price.

### NURSING AT BARNSTAPLE WORKHOUSE INFIRMARY.

THE Barnstaple Board of Guardians lately advertised for a nurse. They received only one application, and the applicant, instead of accepting the salary of £27 they offered, asked for £40. To this they acceded, and the nurse came. But on her arrival she was disillusioned. She had accepted the appointment believing that she would be a superintendent nurse in a country workhouse with four or five nurses under her, probably, to train. The actual experience was that she found herself the only nurse on the female side, and as a trained nurse she did not like to feel that her patients were left to the care of an unprofessional woman at night. She put it to the guardians that the salary and advertisement were somewhat misleading and resigned. Moreover, she was selected for another post which she had declined. The board allowed her to leave at once, and granted her a sovereign towards her expenses. They have now decided to ask the Wootton Association to supply a nurse at £30 a year.

### A COTTAGE NURSING ASSOCIATION IN FINANCIAL DISTRESS.

THE financial state of the Essex County Cottage Nursing Association is very unsatisfactory, notwithstanding the liberal support given to it by Lord and Lady Rayleigh. In presenting the report at the annual meeting, Dr. Thresh said that year by year the deficit increased, and that it now amounted to nearly £150. No pretence is made that the nurses employed by the Association are fully trained; but it is possible that more subscribers might be obtained if a higher standard of training than six months could be introduced. Considering that there are 38 branches of the Association in the county, of which it is affirmed that they are all doing excellent work, the sum of £133 19s., which represents subscriptions, donations, and church offerings for 12 months, is very small.

### NURSING AND MIDWIFERY.

A FEATURE of the fourteenth annual report of the South Wimbledon District Nursing Association is the reference to the practical effect of the Midwives Act of last year. In order to assist in the supply of duly qualified midwives the committee of the association decided to take larger premises with accommodation for a few candidates for certificates who have to supplement their theoretical course in London by some practical experience. They were enabled to venture on this responsibility in consequence of the substantial sum resulting from the Table Tennis Tournament last year. The proceeds of the tournament, which were handed to the association, exceeded £45. We have no doubt that many other nursing organisations will recognise the importance of doing their utmost to assist in the multiplication of trained midwives.

### SHORT ITEMS.

THE Incorporated Society of Trained Masseuses will hold their next examination on June 23rd and 24th. Intending candidates should send in their applications to the Secretary, 12 Buckingham Street, Strand, W.C., before June 2nd, after which date the list will be closed.

### The Nursing Outlook.

"From magnanimity, all fear above;
From nobler recompense, above applause,
Which owes to man's short outlook all its charm."

### DR. DOWNES AND INFIRMARY NURSING.

At the West Midland Poor-law Conference at Malvern last week Dr. Arthur Downes, of the Local Government Board, made the best apologia possible for the late Report of the Departmental Committee on Workhouse Nursing. But the discussion which followed bore strong evidence of the fact that the authorities in general are strongly against the qualified nurse and very doubtful about the working out of the rules for the superintendent nurse. We gave a full report last week of Dr. Downes' paper, and it is only fair now to refer to the comments which that paper called forth, for every day it becomes more evident that the Guardians are adverse to some of the decisions of the Committee, and that the nursing world is very anxious and indignant about the proposed one-year "qualified" nurse. We have struggled so long and so hard for the rise and organisation of the nursing profession that it seems specially bitter, just when victory and peace seemed near, to have the Departmental Committee give a backward push to the movement. It is positively absurd to have Committees sitting to draft Bills for the registration of nurses at the same time that a Committee of the Local Government Board have reported in favour of lowering the term of training to one year in a small infirmary; the last thing we wish to see is the official hall-mark given to the half-taught nurse. And this is evidently the opinion of Miss Gibson, of the Birmingham Infirmary—one of the most capable and successful trainers of nurses for Poor-law work. She said in reply to Dr. Downes that she considered it impossible to train a nurse in an institution of from 60 to 100 patients, for how could a man call a nurse a trained nurse if she had only nursed certain cases? And if a nurse began by nursing old people she began at the wrong end. Incidentally, also, Miss Gibson spoke up for the women guardians, who had been attacked; she said that she had worked with them for 14 years, and she hoped there would always be women guardians. It is probably because the women are standing up so strongly against the "qualified" nurse that they are at present so much out of favour with the central authority. Miss Woollam, of Bristol, asked how the public were to distinguish between nurses trained in the major and the minor hospitals. One year's training was quite insufficient to produce a good nurse; three years had hitherto been found necessary to train a nurse capable of even carrying out doctor's orders. The same good nursing was required for the poor, and she did not think they would get it as a result of one year's training. At the end of a year nurses so trained would be likely to go into the more interesting private nursing, and how was the public to be warned of their inefficiency?

There was absolutely no one to stand up for the one-year's training, but there was one defendant (Mr. Daniels, of Stoke-on-Trent) of the superintendent nurse. And this question of the appointment of the head nurse by the Local Government Board is one which must not be lost sight of because there are other questions to the fore. Mrs. James, of Warwick Union, put the pith of the matter very succinctly: she said that they might expect that the next step the Local Government Board would take would be to appoint a nurse at each and all the unions. Was it desirable to have a superintendent nurse in all unions, when these would be simply the servants of the Local Government Board and not be under the control of guardians? If so, they would be trying to support a very expensive thing.

The Rev. A. G. Burton, of the Atcham Board, remarked that since the Nursing Order of 1897 they had had seven superintendent nurses, and that one nurse had told them she was not under the guardians and had ignored their authority entirely. He did not believe in dual control. From all of which we hope that Dr. Downes and his superiors will see that if "reforms must progress *pari passu* with the march of public knowledge and sentiment," then the time is certainly not ripe to try and thrust the so-called "qualified" nurse on the public and the dual control on the infirmary.

But under all this the opponents of Dr. Downes see and feel the iron hand which is working towards the destruction of all smaller governing bodies—which has already swept away the School Boards, and which aims at the downfall of boards of guardians. The old cry of "local control of local work" is dead, and the new political panacea is one governing body and central control. That is where it is contended we are being rapidly rushed, and once reached it is the ruin of the nursing profession. Further, it is the women who know more about, and feel most keenly, the need for the thorough training of the nurse; and women have no place on the county and borough councils, and when the Poor-law work is relegated to a committee of the county council, then the woman guardian, who has done such good work in the past, will cease to exist. That is the nursing outlook, so far as infirmaries are concerned, from the point of view of the Conference and it is not a cheerful one. All the more honour to women like Miss Gibson and Miss Wilson, real leaders of all that is best in the nursing world, who are doing their utmost to stem the backward movements of their profession.

### Lectures on Medical and Surgical Nursing.

By John Hopkins, F.R.C.S., Medical Superintendent of the Central London Sick Asylum District Asylums, Cleveland Street, W., and Hendon, N.W.

### LECTURE VIII.—DISEASES OF THE NERVOUS SYSTEM.

*Cerebral Hæmorrhage.*—A diseased blood-vessel running through the brain gives way, and blood is poured out into the brain-substance. If the vessel be very small the effect upon the patient will be correspondingly slight; it may amount to little more than a feeling of giddiness. If the vessel be more considerable in size the blood may be in such quantity as to compress the brain and suspend its functions in great part, so that the patient becomes deeply comatose. Coma is a condition of profound unconsciousness in which the breathing is deep and noisy, or stertorous, as it is called, and no movement can be excited in the patient. Hæmorrhage into the brain producing this condition is known as apoplexy. There are all degrees of loss of function between the two extremes of momentary giddiness and coma, according to the degree of compression. Patients generally recover when the hæmorrhage is moderate in amount, but there is permanent loss of function in proportion to the degree of damage to the brain-substance. Hemiplegia is the resulting condition generally. Sometimes there is loss of feeling, but this is nearly always less than the paralysis of movement. This is because the blood-vessels usually give way within the big bundle of motor fibres that carry motor impulses from the convolutions down through the spinal cord to the muscles of the limbs. Cerebral hæmorrhage sometimes causes convulsions. If in the course of your duties in the wards a patient falls in a fit, and you know him to be an epileptic, you proceed at once to deal with the case; but do not let this lead you to assume that when a man has a convulsive seizure it must necessarily be epilepsy. If there be any doubt at all in your mind —if, for instance, the patient is not an epileptic—it becomes your duty to summon medical aid. In dealing with a case of this kind pending the medical officer's arrival do as little as possible to disturb the patient. Place him in a comfortable position and put a pillow under his head, but do not move him further than this, and refrain from all attempts to arouse him, for they only tend to increase the hæmorrhage. The hemiplegia which results is always greater at first than it eventually proves. When the pressure upon the undamaged brain-fibres is removed by the absorption of the blood, these regain their functions, and there is partial recovery. There is generally some stiffness of the muscles at first, called "early rigidity," which passes off after irritation of the brain ceases; and later there is a return of this, called "late rigidity," which causes the limbs to be bent more and more as years elapse, and there is a tendency to fœtor in the palm and between the fingers of the rigidly flexed palm, so that this requires special attention.

Hemiplegic cases tend to move towards the side of the bed that is on their unparalysed side and may fall out, so they should be placed more to the other side. When sensation is partly lost there is in some cases as much tendency to acute bed-sore as there is in paraplegics, of which you will hear something presently. It is on the paralysed side that the bed-sore forms quickest, and this is the side they are inclined to lie upon. In course of time there is rigid acute flexion; the muscles may waste without any relaxation of the limbs which have become fixed by fibrous bands, and sores are to be feared on all points of pressure, especially as the patient has acquired the habit of lying in one particular posture, all others causing pain and discomfort. The incontinence, which is the necessary accompaniment of this condition, soddens the skin, which needs to be frequently attended to in the manner that you have learned to deal with it practically in the wards.

*Paraplegia.*—Hæmorrhage into the substance of the spinal cord may be the cause of paralysis of both lower limbs, and, if it occurs in the neck region, of the upper limbs as well; but paraplegia generally is the result of a fractured spine; of angular curvature, in which abscess forms and compresses the cord; of some morbid growth, or transverse myelitis, in which a short length of the cord is inflamed in its whole breadth. In paraplegia there is loss of sensation as of movement, and loss of power over the bladder and rectum, loss of power to void and to retain. At first there is generally retention of urine, and as there is loss of sensation at the same time the patient has no conscious desire to pass urine. It is in these cases of paraplegia where sometimes the retention comes on unperceived by the patient, and may be one of the early signs of the onset of the paralysis, that the active observation of the nurse may prove of real value. If the patient has not passed urine as usual, her suspicion should be at once aroused. When the urine has been allowed to accumulate in the bladder, it forms a decided prominence above the pubes. Here, again, in washing the patient, this can hardly escape an observant eye. In time the bladder begins to overflow; there is incontinence from retention. At frequent intervals there is a small quantity of urine voided, or there is a constant dribble. The sheet requires frequent changing. A nurse should not rest contented as to her patient's condition when the dry sheets have been scarcely put on before they require to be renewed until the doctor has satisfied himself as to the cause of it. The certain result of urine being allowed to distend the bladder for any considerable time will be cystitis. Within a day or two blood will begin to flow pretty freely from the congested lining of the bladder, and the urine will become ammoniacal. Such a condition adds very materially to the danger the patient is already exposed to.

It is in paraplegia there is such danger of acute bed-sore forming. Having loss of sensation they have no desire for change of posture as healthy people have; no feeling of numbness, pins-and-needles, or aching. Consequently they do not ask for a change of posture, and being powerless they cannot move themselves if they have the desire. Besides that, their own efforts to move may be deterred by pain. So they lie continuously in one position, the weight of their own bodies keeping the capillaries empty in the soft parts rested upon down to the bone. Press one hand upon the back of the other, and see how pale it is on suddenly removing it. If that were kept up for hours the flesh would slough. The part turns purple and then black, and a deep slough of all tissues down to the bone takes place, from the absence of blood to keep the part alive. This may happen within a few hours of the onset of paralysis. One night's neglect to keep the patient moved from one posture to another is enough to cause the worst of acute bed-sores. On the other hand, careful nurses can keep a paraplegic alive for years. Bed-sores form on any point of pressure, the sacrum, the hips, and the heels being the parts most frequently affected. Superficial bed-sores due to wet beds, neglect to change sheets, bread-crumbs, rucks in the sheets, want of due cleanliness and attention to the skin, are specially liable to occur in people who are paralysed and helpless.

In paraplegia, when there is complete loss of voluntary power to move the limbs, they are involuntarily moved by a reflex action through the spinal cord. When the limb is laid hold of it is drawn up. This must not be mistaken for wilfulness on the part of the patient. They are quite unable to prevent the limb behaving in this way. Unless this is understood, people are liable to think that the patient is shamming.

*Spinal Sclerosis.*—When the posterior columns of the spinal cord are hardened or sclerosed by the growth of fibrous tissue the nerve fibres are at the same time destroyed. These are sensory columns of the cord. At the same time there is degeneration of the sensory nerves that pass into the part of the cord sclerosed, and there is loss of sensation. Lightning pains shoot through the limbs, the patient does not know when he is moving his limbs, and they are consequently thrown about as he walks. Hence it is known as locomotor ataxia. He is subject to gastric crises in which there is violent vomiting which may last for a day or two; laryngeal crises, in which he has spasm of the muscles of the larynx, accompanied by a hacking cough and difficulty of inspiration; bladder crises, in which he suffers from retention of urine; and intestinal crises, with diarrhœa. The pains and the various crises can be cut short by medication. The retention of urine requires the use of a catheter. It is in this disease that Charcot's joints, already described, are found, and so-called spontaneous fractures occur.

*Spastic Paralysis.*—In this there is sclerosis of the lateral columns of the spinal cord. Attempts to move the limbs excite rigidity of the muscles, which cause the patient to drag the toes upon the ground. In advanced cases the spasm in the muscles that draw the thighs together, causes the limbs to press so tightly together that deep-pressure sores down to the bone occur on the inside of the limbs.

### Nursing in Workhouse Infirmaries.

At the West Midland Poor-Law Conference last week, the reading of the paper by Dr. Downes on "Nursing in Workhouses and the Report of the Departmental Committee"—of which a summary appeared in our columns last week—was followed by a discussion.

The Rev. A. G. Burton, chairman of the Atcham Union, said that the adoption of the rule of the official committee in their Union had been disastrous, for they had had no fewer than seven superintendent nurses, and now experienced great difficulty in getting nurses. One nurse told him that she was "not under the authority of the Board of Guardians, but of the Local Government Board," and considering their experience at Atcham he thought the Local Government Board should not lay down hard and fast rules, but be content if the nursing was thoroughly efficient. He thought that it was impossible to have dual authority. The master and matron should be the superior officers and the superintendent nurse should be under their supervision.

Mr. Charles Daniels, of the Stoke-on-Trent Union, said that the experience in his Union was exactly opposite to that of Atcham. They had never had any difficulty in getting a nurse, but perhaps that was because Stoke-on-Trent Union was a recognised school for nurses. They had a superintendent nurse who had been with them for 27 years, and a large staff under her. Now that they had a nurses' home they hoped that their nurses would remain a long time with them.

Miss Gibson, matron of Birmingham Infirmary, though congratulating Dr. Downes on his admirable paper, hoped that she might be allowed to combat some of his remarks. A nurse could not be called a "trained" nurse if she had only nursed certain cases; it was necessary that she should have a general as well as a specialised knowledge, which could not be gained in a workhouse where there were 60 to 100 patients. Before a nurse was appointed to any union she should have at least one year in a training school. Her first interest should be that of the sick, and she should have a knowledge of nursing in sickness. How was this knowledge to be gained if she was trained in a small infirmary? With regard to the beginning of her training, if a nurse started by nursing old people she began at the wrong end. As to lady guardians, and the hot words which had been spoken against them, during the fourteen years she had been connected with Boards of Guardians, she had always found them most willing to help her in every way. She hoped that they would continue to have lady Guardians.

Mrs. James, of Warwick, said that they might expect the next step the Departmental Committee would take would be to appoint a nurse at each and all of the unions. Would it be desirable to have a superintendent nurse in all unions when these would simply be servants of the Local Government Board and not under the control of the Guardians? She thought not.

Miss Woollam, of Bristol, asked how the public were to distinguish between nurses trained in the major and in the minor hospitals. One year's training was quite insufficient to produce a good nurse; three years had hitherto been found necessary to train a nurse capable of even carrying out doctors' orders. The same good nursing was required for the poor, and she did not think they could get it as a result of one year's training. At the end of a year nurses so trained would be likely to go into the more interesting private nursing, and how were the public to be warned of their inefficiency?

Dr. Downes, replying to criticisms upon his paper, said that time would not allow of his touching on any but two or three points which had been raised. He was afraid that Miss Gibson had sketched to them an ideal state of things. If she could tell them how to bring it about he thought he might venture to promise her all the help the Local Government Board could give. Personally he could not see the way to giving everybody silver spoons. Some would have to be content with electro-plated ones. There had been, he thought, a good deal of misapprehension as to what were the real recommendations of the committee. Especially had this been the case in regard to the question of authority in the workhouses. He could assure them that the Departmental Committee had not the slightest intention of setting up everybody over one another. They placed the Guardians first, the medical officer second, and strongly recommended that the master should be the head of the institution. Then came the superintendent nurse. He was sorry that she should be put above the matron, but there was really nothing in that, and she only happened to come in that order because they were dealing with the sick wards. Another speaker had regretted that recommendations were not optional. As a matter of fact they were neither optional nor compulsory. It was for the Local Government Board to determine how far they should be carried out. One delegate had asked how small workhouses were to be dealt with. The Committee had recommended several ways, one of which extended to three-fourths of the workhouses in the kingdom, that the matron should be the superintendent nurse. The keynote of the Committee's recommendation was the adoption of the conditions. They had not tried to make an iron cap to fit on to everybody's head without any regard to the size of that head. He had been asked by a lady delegate how many beds he considered should be placed under one nurse. The conditions in nursing varied so much in different workhouses that he would prefer not to attempt an answer to the question.

The President tendered the thanks of the Conference to Dr. Downes for his attendance and for his very able paper.

### Asylum Workers' Association.

THE annual meeting of the Asylum Workers' Association was held at the Medical Society's House last week, and was largely attended. Sir James Crichton-Browne was in the chair, and after the ordinary business had been dispatched he proceeded to address the meeting.

Sir James congratulated his hearers on the prosperity of the Association as evidenced by the increased roll of membership, which now amounted to nearly 5,000. The chief object of the Association was to increase the status and emoluments of asylum workers, and Sir James commented with surprise on the case of an asylum where, although the medical men received liberal salaries the nurses were only paid £14 per annum. After touching upon the question of pensions for the workers, which the Association were endeavouring to bring before Parliament, the Chairman went on to speak of the terrible outbreak of fire at the Colney Hatch Asylum in January. Everyone, he said, must have felt a glow of pride when reading of the courage and fidelity to duty displayed by the staff of the asylum on that memorable occasion. The committee of the Association had at first proposed to bestow medals on those nurses and attendants who were most prominent in the work of rescue; but hearing from Dr. Seward that it was impossible to draw distinctions, they had decided to present a brass tablet as a modest tribute to the gallantry of the staff, which would be, they hoped, placed in the entrance hall at Colney Hatch Asylum.

The tablet was then exhibited to the meeting. The inscription is as follows:—

"In appreciative commemoration of the heroic conduct and self-sacrificing devotion to duty displayed by members of the staff of Colney Hatch Asylum in rescuing from the flames 269 lives of patients in peril by fire on January 27th, 1903, this tablet is placed by the Asylum Workers' Association."

The Chairman subsequently drew attention to the Reading Union and to the competition in connection with it held last year, when the first prize for the best answers to questions on "Ivanhoe" was gained by Mr. H. B. Ellis, of the West Riding Asylum, the second by Nurse A. L. Smith, of the Buckingham Asylum, and the third by Nurse E. Scales, of Hanwell. For next year the book chosen was "King Solomon's Mines," and in addition to the money prize, Mr. Rider Haggard had promised a presentation copy of the book containing his autograph.

The presentation of medals for long and meritorious nursing service then took place, and were awarded as follows:—*First Gold Medal*: Attendant H. Marshall, of the Eastern Counties Asylum, 48 years' service; *Second Gold Medal*: Head Nurse Mary Anne Buckle, of the East Sussex Asylum, Hayward's Heath, 43 years' service. *First Silver Medal*: Mr. John Lynch, Cork District Asylum, 33 years' service; *Second Silver Medal*: Nurse Anne Elizabeth Jackson, Bethlehem Royal Hospital, 32 years' service. It was added that Miss Jackson had not been absent from duty for a single day during all that period.

### Wants and Workers.

WOULD any library or institution care for the gift of THE HOSPITAL (unbound) almost from its commencement? Recipient to pay carriage. Address "Matron," Women's Hospital, Shoeburyness.

### Plaistow Maternity Charity and District Nurses' Home.

THE annual meeting of the Maternity Charity and District Nurses' Home was held last week and was very well attended.

The Marchioness of Bristol, who was in the chair, dwelt upon the work done by district nurses amid the rural population and pointed out the laborious nature of the lives they had to lead in consequence of the scattered population and the distances they had to traverse when visiting their patients. Another difficulty which had to be faced by country nurses was the lack of many necessaries such as would be found in most hospitals and in many private houses among the higher classes. For this work no better preparation could be had than a training in the slums; all nurses who were trained at Plaistow learned how to devise makeshifts. The rural population was also poor, and a district nurse could not expect to make a substantial income unless some of the richer neighbours assisted. If the income were not large it was plain that a nurse could not afford to undergo expensive training, which meant the sinking of capital for which she would get no return. The managers of the Plaistow Home held that six months' training was better than none at all, and their great point was that the training was good as far as it went.

Miss Robinson proposed a resolution commending the work done by the home in training women as midwives to meet the requirements of the new Act, and referred at some length to the Midwives Act, which came into force on April 1st. She insisted that it was a matter of the utmost moment that a sufficient supply of properly-trained midwives should be ready to take the places of the untrained women who had hitherto acted as midwives, but who, by the provisions of the new Act, would be prohibited from doing so. When it was borne in mind that more than half the births which took place were attended by midwives without the supervision of a doctor, it would be seen how necessary such a prohibition was. In very poor districts midwives were a necessity, the mothers being unable to afford a doctor. The hospitals were filled with women who were invalided for life from ignorance and harmful treatment at childbirth, and to this cause was due two-thirds of the cases of child-blindness.

Dr. Sanders, who seconded the resolution, mentioned that the report for 1902 showed a total of 3,482 maternity cases attended, which was more than one-third of the whole number of births in the West Ham district. He paid a tribute to the excellent work done by the nurses during the small-pox epidemic.

A vote of thanks having been passed to Lady Bristol, the meeting adjourned to the garden for tea. Among the company present were Lady Selborne, Lady Ebury, Lady Katherine Drummond, and the Bishop of Colchester.

### The Open-air Treatment.

WE are glad to hear that the attention we have called to Mrs. Mary White's new home for these cases, Bronwylfa, Penmaenmawr, has met the requirements of some of our readers. The demand for this class of accommodation is so great that we should advise our readers to place themselves in communication with Mrs. White without delay, as there seems every probability that her accommodation will soon be taken up for some time to come. Full particulars of this home will be found in THE HOSPITAL of the 16th instant, *Nursing Section*, page 85.

### A London School Nurse's Day.

I start out for a day's work, armed with a few simple dressings, and reach my first school about 9.30 A.M. In the room allotted to me I generally find a boy or girl, who is proud of being called "Nurse's boy or girl," waiting for me with basins and hot water. My young helper goes to each class and informs the teacher of my arrival, and then the children are allowed to come to me. They are a queer little crowd, very dirty, and many of them very ragged, boys, girls, and infants too often showing how little care is bestowed on them, with just a few tidy and clean ones here and there. We divide them into groups, and soon there is a row with shoes and stockings off waiting for toes and other sores to be dressed. While the dressings are going on I show them the wonderful effect of a little soap and water, and teach the elder ones to dress their own wounds. Many of them are most interested, and very proud to come and show me the next week how well they have managed.

Then eyes, ears, and heads have their turn, and I go on to the next school, which is a "Special School for Mentally Deficient" children. Here the dirty children have disappeared, for these youngsters are bathed twice or three times a week at school, and in consequence are better in health and do not require many services at my hands, but there are always some patients, for otitis is one of their troubles. Next I visit a Blind School and here too the children are cared for physically, and I have only a few head cases. So ends the morning's work. Then, after lunch, two more schools fill up the afternoon, and I frequently finish by taking a child or two home and persuading the mothers to have their hair cut or to take the little one to hospital. School nursing is slow work and needs patience. But the results are cheering and the knowledge that the babies are helped and the elder girls taught their first lessons in personal hygiene so that they may make good mothers some day, helps one to go on hopefully.

### Everybody's Opinion.

[Correspondence on all subjects is invited, but we cannot in any way be responsible for the opinions expressed by our correspondents. No communication can be entertained if the name and address of the correspondent are not given as a guarantee of good faith, but not necessarily for publication. All correspondents should write on one side of the paper only.]

### SOLUTION FOR SOAKING SHEETS.

"The House Governor of the General Hospital, Birmingham" writes: I think "Australian Matron," who asks a question in your issue of May 9th, will find a 1 per cent. solution of "Chloros" the most economical and practical medium for soaking "typhoid linen." It is found to be quite effective on bacteriological examination, and has been substituted here, on the advice of our medical committee, on account of the difference in cost. A 5 per cent. solution is used for disinfecting stools, urine, and utensils. The cost of the solution 1 per cent. is about one twenty-fourth of carbolic acid for the linen and one-fifth for the stools, urine, utensils, etc. The matter having been carefully gone into here, our experience may be of service to the inquirer. The solution mentioned (1 per cent.) is found not to be destructive of linen. "Chloros" can be obtained from the United Alkali Company, 30 James Street, Liverpool.

### UNANSWERED LETTERS.

"An Invalid" writes: "A Nurse" complains that her letters in reply to advertisements are unanswered, but there is another side to the question. I advertised for a qualified and experienced nurse, giving the fullest possible details to prevent waste of time and unsuitable applications. That my advertisement was seen is proved by the shoals of letters received, but out of the whole only one was in the remotest degree likely, and in this one the particulars given were so meagre and incomplete as to necessitate writing, asking further details before an interview. This letter has remained unanswered now for a week. The remaining pile must, in the majority of cases, have been written with the full knowledge that they were unfitted for the position and were really not entitled to any reply, whether a stamp was enclosed or not. I replied to all, but I certainly should not trouble to do so again.

### THE OUTCRY AGAINST OUT-DOOR UNIFORM.

"Trained Nurse" writes: Seeing a "note" on this subject in your columns, I should like to say how objectionable it is for nurses to know that many women going about in uniform are not nurses at all, to say nothing of the number of nurse-maids who adopt this style of dress. I think it is the behaviour of these persons that gives many people such a bad opinion of nurses and generally degrades the profession. Can nothing be done to protect the uniform? Supposing nurses were all obliged to register like medical men, and any person wearing uniform unless registered to be liable to punishment. I don't see any reason why it should be discarded, but I certainly think it should be protected. How is it that no other uniform is abused in this way? It is a great convenience, especially to private nurses, who would frequently forego their short daily walk if they had to change before going out and again on returning.

### THE PROBATIONER PROBLEM AT POPLAR.

"Josephine Molony" writes: May I (an old Poplar and Stepney nurse) give expression through your columns to the regret which I, in common with many other nurses who were fortunate enough to be selected and trained by Miss Hannaford, feel at the present unpleasant crisis in the affairs of the infirmary—that infirmary which she by years of indefatigable labour, in co-operation with Dr. Spurrell, has changed from a very ordinary "workhouse infirmary" into a first-class practical training school. When Miss Hannaford first went as matron to the Poplar and Stepney Sick Asylum, she found there a work to be done which only a strong-minded, capable, energetic woman would have dared to undertake, and with which she could not have persevered had she not been actuated by a keen and unselfish interest in her profession. That Miss Hannaford persevered successfully, her nurses in positions of trust and responsibility in hospitals and infirmaries in England and abroad, give ample proof. It is surely much to be regretted that the managers should use to the detriment of an excellent training school, that power which is entrusted to them for its welfare and advancement, and that they should insult, as by their recent measures they unquestionably are doing, a matron whose true worth, business acumen and zeal for her profession have been conspicuous during her whole career.

### TO PREVENT DRAW-SHEETS FROM WRINKLING.

"E. N." writes: I think that nurses in charge of bed-ridden and helpless patients would find "R.B.'s" draw-sheet rather a complicated matter. 1. Whenever the draw-sheet required drawing, which is frequently with bed-ridden patients, it would take some time to untie strings and take out laths. 2. A clean draw-sheet or rather small sheet would be required every time it was drawn. And that would mean at least two or three times a day. 3. In private or hospital work it would be rather an expense, as so many sheets would have to be made both for the exact size of the bed, and also the number which would be used. The draw-sheet used in our hospitals is much more sensible, and if the bed is properly made, and the draw-sheet pulled tight and well tucked in wrinkles seldom appear before the time for drawing, which is at least three times a day, as the patient by then is hot, and glad to have the sheet drawn without any trouble, and then feels cool and refreshed.

### WORDS OF ADVICE TO NURSES.

"M. M. B." writes: Having read the contribution by "The Hermit Crab" in your issue of April 25th, I should like to say how thoroughly I endorse her opinions with regard to Miss Young's papers. I have always felt that a nurse should, for the time being, be able to throw off thoughts and worries of hospital life while not on duty, and that she will find this capacity a distinct gain in every way—in her renewed energy for work, in the added cheerfulness of her face and voice, which will unconsciously be communicated to her patients. She will find it also in her social self—for surely even a nurse has some social duties to perform—which begin to seem irksome if neglected for long—and in her intellectual self likewise. The talents which a nurse possesses are often lost or forgotten during the trying time of her probation, and speaking from personal experience, I know that will be regretted later on. All hobbies or recreations which do not interfere with duty should be encouraged as tending to widen one's ideas and assist the growth of individuality. Women will make finer, more admirable nurses by developing other faculties, and by taking a lively interest in all that goes on in the outside world. I have even met nurses who had become so narrowed down to hospital life, that it required quite an effort to induce them to take an interest even in their own home relations. Respecting outdoor uniform, I do not approve of it, for the reasons given in your correspondent's letter, and I consider it the duty of every woman, nurse or otherwise, to make the best of her appearance. To be careless of it shows lack of self-respect. With all due humility I hope, and think, that my opinions are shared by very many present-day nurses, and that the profession has nothing to lose by them, but something to gain. Finally, I think, there is a large amount of wisdom contained in the old nursery proverb—"All work and no play makes Jack a dull boy."

### SUPERINTENDENT NURSES AND THE MIDWIFERY CERTIFICATE.

"Another Superintendent Nurse" writes: For some weeks I have been following with great interest the letters in The Hospital on the report of the Departmental Committee on Infirmary Nursing, and cannot at all agree with the letter of a "Superintendent Nurse" regarding the maternity certificate. Surely "Superintendent Nurse" does not suppose the L.G.B. intend to appoint superintendent nurses with a maternity certificate only. As I read the report the maternity certificate is to be a compulsory addition to that of general training. A very different thing. I am aware that many have gained their L.O.S. in the manner described by "Superintendent Nurse," viz., by merely seeing the requisite number of cases and reading up. (Is this not a point which might be profitably considered by the Central Midwives' Board). As an experienced superintendent nurse I may say that, after general training, the usual course is to enter lying-in wards as a pupil nurse and train thoroughly in every detail of maternity work. Surely a most important thing when one considers the very large number of lying-in cases passing through our workhouse infirmaries. Even in the smallest, and here there is valuable teaching material which is not utilised unless the superintendent nurse herself is well grounded in the work. If the new order is passed in its present form—and so far as it concerns superintendent nurses everyone hopes it will—it will be more important than before that they should be well trained in every branch of workhouse nursing. It is not the want of a maternity certificate that prevents trained nurses from taking posts under the Poor Law, but their positions under the master and matron and the numberless petty annoyances they are subjected to. While admitting that some workhouse matrons are trained nurses—as at Warwick—they are few in number compared with the large majority who are untrained, and have many of them been appointed from very subordinate positions, and it is with these, who are even now so jealous of the limited authority of the superintendent nurse, that the differences and disputes arise. I am perfectly certain there will be no lack of candidates for superintendent nurses from fully-trained nurses when once their position is properly defined.

### THE ONE-YEAR NURSE.

"Mrs. Richmond, Matron, Luton Union," writes: In a recent issue of The Hospital it was stated that there is an overwhelming preponderance of opinion in the nursing world against the creation of the one-year nurse. I think such is hardly the case in that section of the nursing world with which the one-year nurse is mostly concerned. I do not purpose defending the term "qualified," but with all deference to the signatories of the recent Memorial to the Local Government Board, and at the risk of incurring their displeasure—I unhesitatingly say—that a very good case has been made out for a trial at least of one-year trained nurses. If the representatives of the overwhelming preponderance of opinion had been able to place before the Departmental Committee any practical scheme for providing in the non-training Poor Law infirmaries an adequate staff of fully-trained nurses, the committee and everyone connected with small workhouses would only have been too pleased to give the scheme and the propounders the deference which would then have been legitimately due to them; but it is one thing to bring overwhelming adverse criticism to bear upon the scheme and another to supply a practical solution to the difficulty as an alternative. With regard to objection (*a*) in the memorial, no one will deny that there is a smaller percentage of acute than chronic cases. Still, it must be remembered that it is subordinate nurses which the plan is designed to supply, not superintendents; and the nurses will at least have had 12 months' systematic training according to a syllabus laid down by the Local Government Board in the particular kind of duties which they will ultimately have to perform. This one year's training is designed to bring about (and, I believe, it would to a great extent do so) a uniform standard of efficiency in all subordinate nurses. With reference to (*b*) and (*c*) I would ask, have any of the memorialists an overwhelming preponderance of knowledge (gained from practical experience) of the facilities for training in some of the smaller workhouses? I should say no, or they would not have given such reasons for taking exception to the recommendations; then, lacking practical experience, they cannot expect their word to have the same weight against the proposal as it might have had, had there not been so much evidence to show that in many small infirmaries good training can be given where at present no systematic training is organised; that some such is absolutely necessary to replace the present untrained assistant nurse. On the other hand there was a great deal of evidence from witnesses with practical experience and knowledge of the needs of small workhouses, and the committee no doubt could see that in dealing with the latter it would not do to rely too much upon the advice and opinion of those who were experts only in the administration of large workhouse infirmaries. Now coming to the last objection (*d*), if some of these nurses do take service in private nursing homes, I cannot see how that can be worse than the present state of affairs. Nurses are already taken who have received no systematic instruction whatever, but simply have a smattering of knowledge picked up here and there, so how can the danger to the public be greater in being nursed by women who have received a year's training, proved their efficiency, and obtained a certificate, than by those who come under the former description? But any such danger can be avoided, to a great extent I think, by the medical profession. If a patient requires a nurse he usually requires a doctor, and such doctor knowing the needs of the patient and the difference in the skill of a one-year and a three-year nurse, can state plainly which it is necessary for the patient to have, and if at any time a one-year nurse is passed off as a three-year nurse proceedings should be taken against the nursing home, or, if not too late, the employer should refuse to pay the fee. I believe that many of these nurses will subsequently want the three-years' certificate. Then there will be an opportunity for all matrons who have a sincere desire for nurses to possess it, to show their practical sympathy with the difficulties of the smaller infirmaries by according the nurses all the help they can in that direction; in other words, opening the doors of their training schools and allowing the nurses to enter and finish their professional education. I trust the Local Government Board will reserve to themselves the right at all times to determine whether or not any infirmary shall be recognised as a training school. It has been pointed out with a great amount of truth, that a change of superintendents or medical officers may make a considerable difference in the value of the training. In conclusion, I repeat that I think the plan deserves a trial, and that everyone whose duty it will be to administer any part of it, should do so with the same spirit in which it was evolved, viz., to secure the greatest good for the greatest number.

### Appointments.

Antivivisection Hospital, Battersea Park, S.W.—Miss A. Byford has been appointed matron. She was trained at the Oldham Infirmary, Lancashire, and has since held appointments as head nurse of the male wards at the District Infirmary, Ashton-under-Lyne, night superintendent (*pro tem*) of the Royal Victoria Hospital, Bournemouth, and sister of the Children's Hospital, Torquay (holiday duty). She has for several years been employed in private nursing.

Bedford Union Infirmary.—Miss Kate Girdler has been appointed superintendent nurse. She was trained at the General Infirmary, Northampton, and has since been charge nurse at Mill Road Infirmary, Liverpool, and district nurse at Market Harborough.

Derbyshire Royal Infirmary.—Miss Margaret Elevin Sparshott has been appointed matron. She was trained at Nottingham General Hospital, where she was appointed ward sister. She has also been night superintendent and assistant matron (holiday time), at Birmingham General Hospital, and matron of Grimsby Hospital.

Exeter Infectious Diseases Hospital.—Miss Marian Jones, the newly-appointed matron, was formerly night superintendent and senior sister at the Borough Hospital, Croydon.

Keighley Union Infirmary.—Miss Minnie Sprenger has been appointed charge nurse. She was trained at Barnhill Poor House, Glasgow, where she was afterwards staff nurse. She has since been attached to the Kent Nursing Institution.

Middlesbrough Sanatorium.—Miss M. Webb has been appointed matron. She was trained at Richmond Hospital, Dublin, and has since been sister and night superintendent at the City Hospital, Park Hill, Dingle, Liverpool.

Norfolk and Norwich Hospital. — Miss Bessie Coleridge Davis has been appointed lady superintendent. She was trained at King's College Hospital, and has since been night superintendent of the same institution, and of St. George's Hospital, London; matron of Bromley Hospital; matron of the cancer wing of Middlesex Hospital, and matron of Gravesend Hospital.

Plaistow Hospital.—Miss Rose Hall has been appointed sister of the enteric wards. She was trained at the London Hospital and at the Cancer Hospital, Fulham Road, S.W.

Royal Mineral Water Hospital, Bath.—Miss M. Griffith has been appointed matron. She was trained at Manchester Hospital for Sick Children, Pendlebury, and British General Hospital, and has since been night superintendent at Bootle General Hospital, and lady superintendent of Denbighshire General Infirmary.

Sheffield Union Infirmary.—Miss Clara Appleton has been appointed sister. She was trained at the Sheffield Union Infirmary, and has since been staff nurse and sister in the same institution, leaving last November through ill-health.

Stroud Hospital.—Miss A. E. Forster has been appointed charge nurse. She was trained at Sheffield Royal Infirmary.

West Ham New Infirmary.—Miss E. Gertrude Hobbs has been appointed night superintendent. She was trained for three years at Guy's Hospital, has since been staff nurse at the Royal South Hants and Southampton Hospital, charge nurse at the Northern Fever Hospital, Winchmore Hill, sister at the London Female Hospital, Harrow Road, and sister at Lewisham Infirmary. She has the L.O.S. certificate.

Worcester City and County Institution for Trained Nurses and Spine Hospital.—Miss Annie Michie has been appointed lady superintendent. She was trained at North Lonsdale Hospital, Barrow-in-Furness, and has since been county superintendent at the Nurses' Home, St. Austell, Cornwall.

### TRAVEL NOTES AND QUERIES.

Guernsey Lodgings (Nurse R.).—Many thanks for valuable information which is most acceptable.

Knokke in Belgium (M. A. N.).—The name of the hotel is Hôtel de Bruges, and the name is pronounced as if the last letter was "y." I think you had all the information I could give in that article. Since then (three years ago), prices have gone up a little.

France on £5 (Margaret).—Dieppe will not do, it is expensive. I give you a choice of three places. Caen, Caudebec on the Seine, Rouen itself, or St. Servan in the Bay of St. Malo. Either of these places you can stay in and pay your journey for £5 with care. Tell me which you favour, and I will give you addresses, but space is precious. If you like to go to a convent quite in the country I could tell you of one remarkably cheap. I could also tell you of cheap residence in Bruges, and the journey would be much cheaper.

Lausanne and Chamonix (A. M. R.).—Certainly go independently. Conducted parties are for the helpless; your means are ample. At Lausanne go to Hôtel Pension Victoria, Avenue de Rumine: pension from 6 frcs. per day; or Hôtel Pension du Village Suisse, which is up high. This last is extremely comfortable. At Chamonix Grand Hôtel Couttes, from 8 frcs. (this is very English), or Hôtel Beau-Site, rather at the end of the village, from 6 frcs. One mile from the village, Hôtel Pension du Lac, about the same. You will probably go to Zermatt, Hôtel Mont-Rose, 7 to 16 frcs. If you wish to visit the great St. Bernard, which is easily done from Martigny, you will lodge at the Hospice itself—a most interesting experience.

A Long Voyage for an Invalid (Med. Student).—The best line I know of for your purpose is the "Loch Line" from Glasgow to Adelaide and Melbourne, and on to New Zealand if desired. First-class return, £76; this includes everything, and, being sailing ships, the time occupied to and fro would be about six months, so that the passenger is really boarded and lodged for nearly half a year. There are other lines where for a very small sum passengers are taken; these ships are called "tramps," and sail from port to port with fruit, etc. . . . but they naturally take no stewardess, and are fit only for the fairly robust. If I can help further let me know. I could give you addresses of some of these latter lines, but they make it rather a favour. I could, I think, give you an introduction, but remember it is rough.

Dr. Lunn's Tours (A. A. H.).—No, you certainly could not do the trip, independently, for anything like the same price. It is co-operation with the railway authorities that contrives this phenomenal cheapness. The cheapest ticket (taken alone) to Chamonix, return, is £5 12s. 4d. Yes; Geneva is quite equal to the neighbourhood of Lucerne, but very different. I cannot recommend any pension in Paris at terms so low as you speak of. The cheapest I know is Mme. Ledoux, 20 Rue Clairant, Avenue de Clichy, 35 francs per week. Next, Miss Bromhead, 47 Boulevard Gouvion St. Cyr. There was a short while since a Miss Worthington, 4 Rue des Pyramids. These pensions are constantly closed without much warning, so it is as well to write beforehand. The terms you mention are really impossible to be thoroughly clean and respectable. You know living in Paris is as dear as London.

Paris and Antwerp (M. E. B.).—We do not reply by post unless 2s. 6d. is sent for our Convalescent Fund. Let me beg you to think seriously before you seek work in Paris. Unless you have introductions and some influence, I fear you have but little chance. You cannot make so expensive a journey as you propose, and remain abroad for three weeks on £10. That is quite impossible on the lines necessary for you. It could only be done by living in some quiet country spot. It seems to me a bad plan to try and do Paris and Antwerp at the same time. If you seek a post in Paris you might first write to the Hertford Hospital, and ask if they would have a vacancy in February. A reasonable and respectable hotel there is Hôtel Britannique, 20 Avenue Victoria. Rooms on fourth floor and on fifth, 1 franc 50. At Antwerp, go to Hôtel du Commerce, 10 Rue de la Bourse, pension terms from 7 francs. At Ghent, Hôtel d'Allemagne Marché aux Grains.

Travel Editor.

### Echoes from the Outside World.

### The King and Queen.

Last week the King and Queen returned to London, having worked very hard during the five days of their visit to Scotland. The "Court" at Holyrood was not over till nearly 6 o'clock on Tuesday evening, and on Wednesday at 11, within the grounds of Dalkeith Palace, the King presented war medals to the 1st Black Watch, the 17th Lancers, and a few men of the Royal Garrison Artillery. The presentation was followed by a march past of the troops. Next came an address from the Corporation of Dalkeith. Then the Royal party came in to Holyrood by train, the King inspected his Royal Archers—about 220 noblemen and gentlemen, mostly over 6 feet high—and a large luncheon party was held at the palace. During the afternoon the Royal guests drove through the city, visited the Castle and St. Giles' Cathedral, and went on to Colinton Mains, four miles distant, where, with a golden key, the King opened what is said to be the finest hospital for infectious diseases in the United Kingdom. The Queen planted an English elm in the grounds, which she designated "a bonny tree," and then the King followed suit with a Scottish elm, and shovelled in the earth with right goodwill. Unfortunately, the next day, which was devoted to a visit to Glasgow, was wet, but rather than disappoint the people, their Majesties drove in an open carriage, the King holding an umbrella high over the Queen. They laid the foundation of a technical college, received addresses in the banqueting hall of the municipal buildings, where they sat in the two gold chairs made originally for Queen Victoria and the Prince Consort, lunched with the Lord Provost of Glasgow, upon whom the King conferred a baronetcy, and afterwards visited the Art Galleries and Glasgow University. Upon their arrival in town from the North on Friday their Majesties received a most enthusiastic greeting from their London subjects. On Saturday they visited the Royal Military Tournament at the Agricultural Hall, Islington. On Wednesday the King, who was accompanied by the Queen, opened the new Kew Bridge, which has been built across the Thames at a cost of nearly a quarter of a million.

### Royalty and Trams.

The Prince and Princess of Wales inaugurated the new electric tramway service, between Westminster and Tooting, on Friday, afterwards riding in one of the cars, which had been painted white and festooned with evergreens for the occasion. The Prince stood upon the top of the car, his two sons, Prince Edward and Prince Albert, accompanying him, but owing to the overcast sky the Princess—who looked extremely nice in a Wedgwood blue costume, a toque to match, and a white feather boa—did not leave the interior, temporarily fitted as a blue and white drawing-room. The car was preceded by two pilot cars, and at Kennington the Princess received a bouquet from the May Queen of the Kennington Board School. Upon their arrival at Tooting their Royal Highnesses descended, and Lord Carrington, as Chairman of the Housing Committee, showed them over three of the County Council's working-men's dwellings on the Totterdown Fields Estate. The rents vary from 6s. to 12s., and though the rooms are very small, the exterior is tasteful. The Princess, she said, was pleased to note that in accordance with the Queen's suggestion at Millbank, cupboards had been put into the cottages for the convenience of tenants, but suggested that the kitchens lacked shelves. These, Lord Carrington promised, should be added. Upon the return journey the Princess, as well as her husband and children, rode outside, and the people, who had densely lined the route all the way were in consequence much gratified.

### The Sailors' Palace.

Within three minutes' walk of Limehouse Station, a very fine institution for the use of sailors in the metropolis was opened by the Prince and Princess of Wales on Tuesday. The Palace has cost £36,000, of which £14,000 has been provided by Mr. Passmore Edwards. The foundation-stone was laid two years ago by the Duke of Fife; there is a navigation school entitled, by command of the King, "King Edward VII. Nautical School," where 40 or 50 students may study for the Board of Trade examinations, and for these students what is practically hotel accommodation is provided. The "Alexandra Wing," named after the Queen, contains the John Cory hall, captains' and officers' rooms, etc. There are numerous bedrooms and a first-class restaurant, also seamen's cubicles to accommodate 30 sailors, and in all about 100 rooms, several endowed by friends. Over the door of one is written, "American ladies equipped and endowed this room"; over another the pathetic announcement, "A sailor's mother out of her poverty gave the first £10"; and over a third, "An English lady gave the site." In the principal hall a series of panels are to be placed in honour of "Humble Heroes of the Sea." After the Prince had formally opened the building, the Princess received contributions, and inaugurated the "Ocean Library," which is a feature of the institution.

### Arctic Geography.

On Monday Admiral Sir Clements Markham presided over the anniversary meeting of the Royal Geographical Society at Burlington House. Of the two events of the year, he stated, one was in the Far North and the other in the Far South. Captain Sverdrup and Captain Peary by their researches had solved the whole problem of Arctic Geography, though there were still several isolated pieces of work to be done. One had been entered on by Captain Amundsen, who was just about to commence his daring voyage in a very small vessel to the North Magnetic Pole. As to Antarctic regions, Dr. Nordenskiöld and his party had been expected to have returned ere this, and a relief ship would have to be sent out to these gallant Swedish explorers if they did not appear soon. The President reminded his hearers that the *Morning* had to be sent out last year to the relief of the *Discovery*, whose sailors badly required assistance. Scurvy was threatening, but the frozen fresh meat brought by the *Morning* at once improved the health conditions. It was not easy work, however, to hand over the provisions for 10 miles of ice separated the two ships, and everything had to be dragged across in sledges. The *Morning* will have to go south again next December, and funds must again be provided.

### Old Embroideries and Antique Lace.

An interesting exhibition was opened at Messrs. Debenham and Freebody's, Wigmore Street, last week, and will remain open till the end of the month. The antique lace is exceedingly fine, and includes old Venetian point, Milanese pillow, Genoese pillow, Flemish, and Brussels, besides many others. The Old Court lappets, which were collected by one of the best judges of lace of the past century, are particularly to be noted. Amongst these is a magnificent specimen of Louis XIV. work in perfect preservation. Specially worthy of mention is an old Venetian altar Frontal of seventeenth century work, the centre representing the Chalice of the Holy Order, surrounded by a wreath of fruit and flowers and supported on either side by figures of angels. It is valued at £500. The embroideries include a large number of pictures in the finest needlework. An oval pair of lady and gentleman archers, are in excellent colouring; another is after Morland, "The Woodman with Dog;" a third, a Stuart picture of two princesses in centre finely worked on cream silk. There is also a map of England and Wales all done in chenille; a view of Esher house with the river, and an embroidered pincushion of Charles I. period, as well as a number of samplers, some lovely brocades and examples of drawn-linen and lacis work.

### For Reading to the Sick.

ASCENSIONTIDE.

'TWAS on the Mount of Olives,
Nigh where the faithful Three
Had bid the Master welcome
So oft at Bethany,
'Twas there the Man we cherish,
The Mighty God we praise
Among His Chosen ended
The wondrous forty days.

There was not ought about Him
The coming change to say,
Only a cloud was o'er them
As on a common day;
He stood with Hands uplifted;
He blessed; that Blessing o'er,
After that earthly pattern
He will not bless them more.

For while He spake, the Saviour
Passed from this world of ill
Far o'er the sacred village,
Far o'er the ancient hill.
Love unto Love returning,
Light to Its kindred Light,
The cloud o'erhead he entered
And passed from mortal sight.

*By A. M. M.*

Believe that heaven has not taken Christ away from you, but brought Him nearer to you; and that He has ascended up on high, not that He, in Whom alone is life, might empty this earth of His presence, but that He might fill all things, not this earth only, but all worlds, past, present, and to come. Believe that wherever two or three are gathered together in Christ's name, there He is in the midst of them; that the Holy Communion is the sign of His perpetual presence; and that when you kneel to receive the bread and wine, Christ is as near you—spiritually, indeed, and invisibly, but really and truly—as near you as those who are kneeling by your side.

And if it be so with Christ, then it is so with those who are Christ's, with those whom we love. It is the Christ in them which we love; and that Christ in them is their hope of glory; and that glory is the glory of Christ. They are partakers of His death, therefore they are partakers of His resurrection. Let us believe that blessed news in all its fulness, and be at peace. A little while and we see them; and again a little while and we do not see them. But why? Because they are gone to the Father, to the source and fount of all life and power, all light and love, that they may gain life from His life, power from His power, light from His light, love from His love.

*C. Kingsley.*

Thou art gone up on high
To mansions in the skies;
And round Thy throne unceasingly
The songs of praise arise;

But we are lingering here,
With sin and care oppress'd;
Lord, send Thy promised Comforter,
And lead us to Thy rest.

*Hymns A. and M.*, 149.

### Notes and Queries.

**REGULATIONS.**

**The Editor is always willing to answer in this column, without any fee, all reasonable questions, as soon as possible.**

**But the following rules must be carefully observed:—**

1. **Every communication must be accompanied by the name and address of the writer.**
2. **The question must always bear upon nursing, directly or indirectly.**

**If an answer is required by letter a fee of half a-crown must be enclosed with the note containing the inquiry. We cannot undertake to forward letters addressed to correspondents making inquiries unless a stamped envelope is enclosed.**

*Maternity Question.*

(70) 1. Has coffee any effect in drying up the milk of a nursing woman? 2. Is it advisable to let a baby sleep without a pillow?—*Baby.*

In both cases you should consult a medical man.

*Medical Profession.*

(71) Will you kindly tell me what age women begin to train for the medical profession, and is it very expensive? Would there be any advantage in going in for nursing first?—*Olive.*

Write to the Dean, London School of Medicine for Women, 7 to 11 Hunter Street, Brunswick Square, W C. She will be able to supply you with the information you require.

*Madras.*

(72) Will you kindly tell me if there are any private nursing institutions in Madras? I am going out to stay with a friend, and am anxious to take up private nursing on my own account.—*Wooley.*

There are no private institutions, but doubtless the Matron of the General Hospital, Madras, would give you information as to private work.

*South Africa.*

(73) Will you let me know of two or three addr esses of nursing institutions and asylums in South Africa as soon as possible?—*Nurse Hermione.*

See "Burdett's Hospitals and Charities" for a full list of the hospitals in South Africa. The nursing institutions at Capetown and Johannesburg have so many applications from nurses that the superintendents are unable to reply to all the inquiries.

*Second Hand Female Pelvis.*

(74) Can you please tell me how or where I could procure a second hand female pelvis and fœtal skull.—*B. J.*

You had better advertise.

*Book on Nursing.*

(75) Is there a book on nursing by the Hon. Sydney Holland, which I believe was published about February 1902? What is the title, and who are the publishers?—*Sister Theo.*

The only book by Mr. Holland is "A Talk to the Nurses of a London Hospital," price 6d., published by Messrs. Whitehead, Moore and Co., Limited, 9 Fenchurch Street, E.C.

*L.O.S.*

(76) Will anyone give me full particulars of the L.O.S. Exam.? I am a stranger in London.—*S. A.*

Write to the Secretary of the London Obstetrical Society, 20 Hanover Square, W.

*Cook's Wages.*

(77) I shall be much obliged if I can find out what wages a cook in an infectious hospital should receive. The average number of patients is 30, and that of the staff 18. No late dinners and a kitchen-maid kept.—*Wondering.*

The advertisements in our columns might be some guide, and the local registry offices should be able to tell you what you ought to pay in your neighbourhood. Wages vary very much in different parts of the country.

**Standard Nursing Manuals.**

"The Nursing Profession: How and Where to Train." 2s. net; 2s. 4d. post free.

"Nursing: Its Theory and Practice." (Revised Edition). 3s. 6d. post free.

"Surgical Ward Work and Nursing." (Revised Edition). 3s. 6d. net.; 3s. 10d. post free.

"Practical Handbook of Midwifery." (New Edition). 6s. net; 6s. 3d. post free.

"Notes on Pharmacy and Dispensing for Nurses." 1s. post free.

"Fevers and Infectious Diseases." 1s. post free.

"The Art of Massage." (New Edition). 6s. post free.

### Travel Notes.

By Our Travelling Correspondent.

### CXXI.—THE GREAT MASTERS OF THE PAST.

### Giorgione.

The romantic personality of Giorgio Barbarelli, better known to the artistic world as Giorgione, must always appeal strongly to the sentiment of the world. Sentiment never dies with us; though it is often hidden under the push and struggle of modern existence it still exists and thrusts an interested and restless inquiry into the romances of the past. Giorgione's irregular birth, the neglect he experienced from the parental side—his early life in the great Bellini's studio—his friendship (interrupted later on by professional jealousy) with Titian, his versatile genius —for he was no mean musician as well as being a giant in his own particular art—his gay pleasure-loving life so soon quenched in silent death—perhaps not least, his great personal beauty and vast stature, which earned him the name of Giorgione (Great George), and lastly his tragic death, when in the flower of his youth, strength, and prosperity, he perished of the plague in 1511, all contribute to make him remembered with fidelity.

### His Birth and Childhood.

It is generally supposed that he was born at Castelfranco, but as his mother was a humble girl of Vedelago, it is possible his birth took place there, though his early life is always associated with Castelfranco. His father was of the Barbarelli family, and by them he was never acknowledged until he became famous some twenty years later, when the noble Barbarelli hastened to claim kinship with him, thus showing that snobbishness existed in the sixteenth century as well as in the present day.

### The Bottega of Bellini.

To Giovanni Bellini's studio, or Bottega as it was simply called in those days, came Giorgione at a very early age, probably when he was about 11 or 12 years old, for it was the custom for great artists to have apprentices, who began their artistic training by grinding colours, washing brushes, etc., for their masters.

Here after a few years he was joined by Titian, who it seems was some four or five years junior to Giorgione. The latter worked very quickly and Titian was a plodder. Messrs. Crowe and Cavalcaselle think that after their apprenticeship to Bellini came to an end Titian became a pupil of Giorgione's; but I do not think that is by any means proved; they were too nearly of an age and very shortly were both engaged as equals on the work of decorating the Fondaco dé Tedeschi. Probably Titian greatly admired his friend's work, and more or less adopted his style of rich colouring.

Giorgione worked rapidly and gave much to the world, but unfortunately a great proportion of his time was given to fresco, and as it was on the exterior of buildings time and weather has robbed us of most of his masterpieces. Titian matured more slowly, as was perhaps natural, for he had 90 years to live, whereas Giorgione burst on the world as a meteoric genius, and as a meteor quickly passed, leaving his gay life, and unfinished work, at the age of 33.

### His Sacred Pictures, Portraits, etc.

As his working life was so short, it is a sure fact that he could not have produced all the pictures attributed to him; probably the larger number were the work of his pupils, who lovingly copied to the best of their ability the style of the man so much beloved, both as a master and a man. There are really very few pictures that can be regarded as without doubt by him. Foremost we must put the altar-piece in the church of San Liberale at Castelfranco. On the back of this panel occur four lines, which read as the sigh of a lover:

"Vieni o Cecilia

Vieni t' affretta

Il tuo l' aspetta

Giorgio "—

Who was Cecilia? we vainly ask. Was she the fair mistress who with her last breath left a legacy of death to her beloved? One would fain think so, but that picture was painted early in his career, and though one does not believe all that the agreeable old scandalmonger Vasari tells us, there is much that makes one fancy Giorgione might be an ardent but not very constant lover. Then his portrait of the Doge Loredano is undoubtedly genuine, and "The Concert" in the Pitti—this latter is a very striking picture of an Augustinian monk playing on a musical instrument to an interested audience. There is another work called "The Concert" attributed to him in the Louvre. It is a sylvan scene, the nymphs have little or no drapery, and the shepherds make up for this deficiency by appearing in parti-coloured tights, hats, feathers, and frippery generally of the sixteenth century.

### The Frescoes on the Fondaco dé Tedeschi.

These, alas! have almost disappeared, the only traces left are on the side facing the canal. It was over these frescoes that Titian and Giorgione fell out. It is an acknowledged fact that artists are an irritable race, and a friend, officious and mistaken, praised Titian's work to Giorgione, imagining it to be the latter's own fresco. A breach was caused, and though the kindly nature of Giorgione would doubtless soon have triumphed over his wounded vanity, untimely death cut him off before the old friends were reconciled. Of these frescoes Vasari speaks thus: "Accordingly Giorgione set to work, but with no other purpose than to make figures at fancy to display his art, for I cannot discover what they mean, whether they represent some ancient or modern story, and no one has been able to tell me. Here is a lady and there a man in various attitudes, a lion's head is hard by, while another has an angel fashioned like a cupid, and I cannot tell what it means. There is certainly a woman over the principal door leading to the Merceria, seated above the head of a dead giant, almost like a Judith. She is raising the head with a sword and speaking to a German below. I cannot explain this in anyway unless he wished to represent Germania!"

The naïve old chronicler is not ashamed to confess his inability to grapple with the ideas of the artist. How well it would be if we moderns would own a like lack of understanding sometimes instead of expressing sham ecstacies.

### His Death.

In 1511 at Venice swift death overtook Giorgione. His mistress died in his arms of plague, and he followed her to the grave in a few hours. All chroniclers seem to agree in the main with this statement, though Ridolphi varies it a little by saying that the lady had first been untrue to him, and the treachery broke his heart and thus rendered him in his despairing state a more easy victim of the plague.

